# Alternative Splicing and Its Roles in Plant Metabolism

**DOI:** 10.3390/ijms23137355

**Published:** 2022-07-01

**Authors:** Pui Ying Lam, Lanxiang Wang, Clive Lo, Fu-Yuan Zhu

**Affiliations:** 1 Center for Crossover Education, Graduate School of Engineering Science, Akita University, Tegata Gakuen-machi 1-1, Akita City 010-8502, Akita, Japan; 2 Co-Innovation Center for Sustainable Forestry in Southern China, College of Biology and the Environment, Nanjing Forestry University, Nanjing 210037, China; amelia0610@163.com (L.W.); fyzhu@njfu.edu.cn (F.-Y.Z.); 3 CAS Key Laboratory of Quantitative Engineering Biology, Shenzhen Institute of Synthetic Biology, Shenzhen Institute of Advanced Technology, Chinese Academy of Sciences, Shenzhen 518055, China; 4 School of Biological Sciences, The University of Hong Kong, Pokfulam, Hong Kong, China; clivelo@hku.hk

**Keywords:** alkaloids, alternative splicing, ascorbate, phytohormones, lipids, metabolism, phenylpropanoids, plants, starch, terpenoids

## Abstract

Plant metabolism, including primary metabolism such as tricarboxylic acid cycle, glycolysis, shikimate and amino acid pathways as well as specialized metabolism such as biosynthesis of phenolics, alkaloids and saponins, contributes to plant survival, growth, development and interactions with the environment. To this end, these metabolic processes are tightly and finely regulated transcriptionally, post-transcriptionally, translationally and post-translationally in response to different growth and developmental stages as well as the constantly changing environment. In this review, we summarize and describe the current knowledge of the regulation of plant metabolism by alternative splicing, a post-transcriptional regulatory mechanism that generates multiple protein isoforms from a single gene by using alternative splice sites during splicing. Numerous genes in plant metabolism have been shown to be alternatively spliced under different developmental stages and stress conditions. In particular, alternative splicing serves as a regulatory mechanism to fine-tune plant metabolism by altering biochemical activities, interaction and subcellular localization of proteins encoded by splice isoforms of various genes.

## 1. Introduction

Metabolism is made up of networks of biochemical reactions that produce a diverse array of organic compounds in living organisms. In plants, metabolism can be categorized as primary metabolism and specialized metabolism. Primary metabolism such as tricarboxylic acid cycle, glycolysis, shikimate, lipid and amino acid pathways generates low-molecular-weight organic molecules directly involved in basic life functions and is essential for growth, development and survival of plants [1]. On the other hand, specialized metabolism, which is also known as secondary metabolism, comprises metabolic processes that are not absolutely essential for growth of plants. Specialized metabolites are natural phytochemicals synthesized from primary metabolic pathways [2] and were once considered as byproducts, waste products or detoxification products of primary metabolism [3]. Well-known plant-specialized metabolites include phenolics, alkaloids, terpenoids, saponins, etc.

In response to different growth and developmental stages as well as for adaptation to environmental changes, metabolism in plants is tightly regulated at different molecular levels. These include transcriptional regulation; post-transcriptional modifications such as alternative splicing, RNA methylation [4], RNA editing [5], and mRNA decay; translational regulation; post-translational modifications such as protein phosphorylation, methylation and acetylation [6], ubiquitination, and protein turnover. Understanding these regulations will establish the foundation for molecular breeding or bioengineering to improve plant growth, yield and biomass production as well as enhance plant stress resistance.

**Figure 1:**
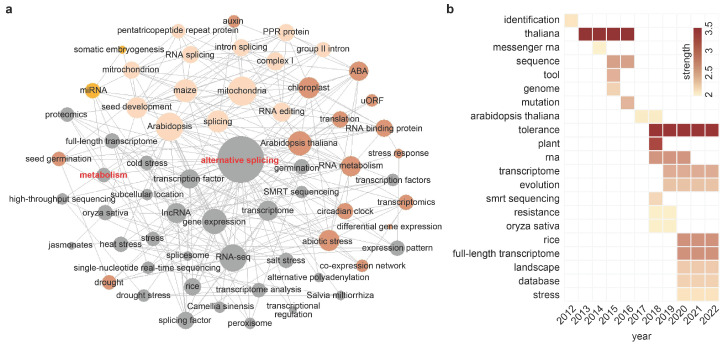
Analysis of keywords in Web of Science Core Collection database from January 2012 to May 2022. (**a**) Keyword co-occurrence network analyzed by BibExcel and Pajek. Nodes represent keywords. Node size represents the frequency of keywords that appear. Node colors represent modularity. (**b**) Burst keyword analysis. Length of colored boxes represents burst status duration. Colors represent burst strength. Bibliometric analysis was carried out by retrieving citation data on topic search using query: “TS = (alternative splicing OR splicing factor) AND plant AND (metabolism OR metabolic OR metabolize)” and was further analyzed by CiteSpace (https://citespace.podia.com, accessed on 1 June 2022).

Bibliometric analyses through keyword co-occurrence network analysis and burst word detection analysis of the Web of Science Core Collection database reveal that studies of alternative splicing in the last decade are mainly associated with various processes and conditions such as stresses, seed development and circadian clock (Figure 1). However, research on the relationship between alternative splicing and plant metabolism has seldom been reported or explored in detail. In this review, we focus on the roles of alternative splicing, an understudied yet potentially important regulatory mechanism in plant metabolism. We first briefly introduce the occurrence of alternative splicing in plants. We then describe and update the current understanding of the roles of alternative splicing in regulating metabolism of starch, lipid, photorespiration, ascorbate, auxin, jasmonates, terpenoids, alkaloids and phenylpropanoids in plants, with specific examples of how alternative splicing regulates gene functions and metabolism (Table 1), and recommend future research directions.

## 2. Alternative Splicing and Its Roles in Plants

### 2.1. Gene Splicing Machinery in Plants

Most plant genes contain introns, which are non-coding sequences interrupting exons that have to be removed from pre-mRNA by a process called splicing. Pre-mRNA splicing is performed and controlled by a ribonucleoprotein complex called spliceosome and other spliceosome-associated proteins [7,8]. So far, two types of spliceosomes have been described [7,9,10]. U2 spliceosomes that are composed of five small nuclear RNA (snRNA) subcomplexes (U1, U2, U4, U5 and U6) represent the major type [7,9,10]. The minor and rare U12 spliceosome also consists of five snRNA, including U11, U12, U4atac, U5 and U6atac [10]. Both types mainly recognize and splice the exon-intron junctions that harbor 5′-GT-AG-3′ sequences [10]. Accordingly, for assembly of spliceosomes, a 5′ splice site with a conserved GT sequence and a 3′ splice site with a conserved AG sequence as well as a conserved A nucleotide at 18 to 40 nucleotides upstream of the 3′ splice site and a polypyrimidine tract after branch point are required [11]. Meanwhile, non-snRNA splicing factors, including serine-/arginine-rich proteins and heterogeneous nuclear ribonucleoproteins (hnRNPs), are also known to assist localization of splicing enhancers and suppressors, thereby regulating the selection of splice sites by spliceosomes [7,12,13]. Numerous studies suggest that mutations of spliceosomal proteins have led to altered alternative splicing as well as growth and developmental defects, including embryonic lethality, abnormal flower and leaf morphology, early flowering, defects in seed maturation and/or hypersensitivity to abscisic acid [14,15,16,17,18]. Apparently, spliceosomes govern accurate splicing, which is essential for normal plant growth and development. During splicing, two transesterification reactions occur, excising introns and joining exons [19]. The detailed process of splicing was reviewed elsewhere previously [7,20].

### 2.2. Alternative Splicing in Plants

**Figure 2:**
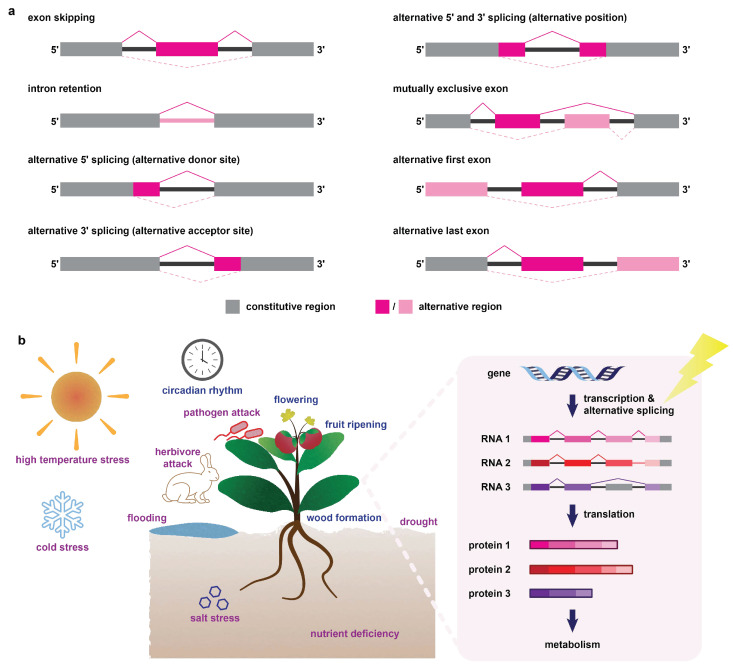
Alternative splicing and its regulation on metabolism in plants during stress responses and development. (**a**) Common types of alternative splicing events in plants. Boxes: exons; horizontal lines: introns; (**b**) regulation of alternative splicing by stresses and developmental stages. Stresses that regulate alternative splicing are indicated in purple. Developmental stages that regulate alternative splicing are indicated in navy blue.

Alternative splicing refers to the generation of multiple splice isoforms (mRNA transcripts) from a single gene due to the use of different splice sites [21]. Such a process largely expands the coding capacity of genomes [22,23] and is believed to be a post-transcriptional regulatory mechanism in response to developmental and environmental changes. The existence of alternative splicing was initially uncovered in some plant species in the early 1980s [24,25,26,27] and was later found to be a common phenomenon in different living organisms, including plants [21]. For example, in *Arabidopsis* (*Arabidopsis thaliana*) and rice (*Oryza sativa*), it was estimated that at least 42% and 48% of intron-containing genes are alternatively spliced, respectively [28,29]. Several types of alternative splicing events have been reported in plants. These include exon skipping, intron retention, alternative 5′ splicing (alternative donor site), alternative 3′ splicing (alternative acceptor site), alternative 5′ and 3′ splicing (alternative position), mutually exclusive exon, alternative first exon and alternative last exon (Figure 2a) [21,22,30]. Among these, intron retention is the most prevalent type of alternative splicing event that takes place in plants [21,28,30].

In plants, alternative splicing is regulated by several factors. For example, the concentration and compositional ratio of splicing factors was suggested to mediate alternative splicing [31]. In addition, structure of chromatin, including DNA methylation and histone modifications, may affect accessibility and elongation speed of RNA polymerase II as well as the recruitment of splicing factors, thus mediating splicing outcomes [31,32,33]. Alternative splicing mediates gene expression or functions at both RNA and protein levels. At RNA level, alternative splicing may affect mRNA stability and result in nonsense-mediated mRNA decay due to frame shift mutations and formation of premature stop codons [30,34,35]. At protein level, alternative splicing may impact translation efficiency [36], subcellular location [37], biological and/or biochemical functions and interaction [38] of proteins translated. Alternative splicing is a regulatory mechanism during stress defense and development of plants [39]. With the development of high throughput RNA sequencing techniques and proteomic approaches such as sequential window acquisition of all theoretical mass spectra (SWATH-MS) and alternative splicing-related bioinformatic platforms such as PlantSPEAD for alternative splicing analysis and validation [40,41], numerous genes in plants have been uncovered to be alternatively spliced when subject to stresses such as pathogen infection [38,42,43], herbivore attack [44], high-temperature stress [45,46,47], cold stress [48], salt stress [49,50], iron and phosphate deficiencies [51], flooding [52,53] and drought [54]. They are also detected in various developmental stages, functioning in the regulation of circadian rhythm [55], wood formation [56], flowering [57,58] and fruit ripening (Figure 2b) [59].

## 3. Current Understanding of the Roles of Alternative Splicing in Plant Metabolism

### 3.1. Overview

Regarding our knowledge on alternative splicing in plant metabolism, recent studies have mostly focused on global evaluation of the extent of alternative splicing, particularly on detecting alternative splicing events and types. From these research studies, numerous genes of plant metabolisms have been reported to be alternatively spliced under different developmental stages and stress conditions. However, the precise physiological and biochemical meanings of the presence of different splice isoforms of these genes have only been partially understood.

### 3.2. Primary Metabolism

#### 3.2.1. Starch Metabolism

Starch is a primary carbon source in human and animal diets [60]. Together with sucrose, starch serves as the major product of photosynthesis and the primary energy storage in plants. Starch is composed of amylose and amylopectin, which are both formed by polymerization of glucose [60]. Starch metabolism, including its biosynthesis and degradation, controls the storage and release of carbon in plants [61]. Thus, starch metabolism defines the quality and yield of cereal grains and other food crops, serving as an important constituent in agriculture and biorefineries.

Cold stresses induce alternative splicing of genes related to starch metabolism. For example, under cold stress, genes related to starch and sugar metabolism in tea plants (*Camellia sinensis*) are alternatively spliced and show correlation with various sugar accumulation, likely conferring resistance towards cold stress [62].

Alternative splicing alters subcellular localization and catalytic activities of starch-branching enzyme (SBE), which functions in determining chain length and branch point frequency of amylopectin in *Phaseolus vulgaris L*. [37]. This is achieved by using alternative first exons and translation start sites of the pre-mRNA of SBE for protein translation [37]. Consequently, an isoform (LF-PvSBE2) harboring an N-terminal plastid targeting sequence that is targeted to both starch granule and cytosol and another isoform (PvSBE2) with a truncated N-terminus that leads to cytosolic localization are generated [37]. Interestingly, the altered N-terminus also affects substrate binding affinity and catalytic efficiency of the enzyme [37].

Alternative splicing has been found to mediate starch metabolism in plants by controlling the expression abundances of different splice isoforms of transcriptional factors. For example, in *Arabidopsis*, the transcription factor indeterminate domain 14 (IDD14) mediates starch accumulation by activating *Qua-Quine Starch* (*QQS*) [63], which is involved in starch degradation [64]. Under cold stress, a splice isoform IDD14β that lacks DNA binding domains was induced [63]. IDD14β binds to the full-length functional isoform IDD14α and inhibits its DNA binding ability, resulting in reduced transcription of *QQS* and altered starch accumulation [63]. On the other hand, rice *OsbZIP58*, a basic leucine zipper transcription factor that is highly and specifically expressed in endosperm, mediates grain filling by regulating the expression of starch biosynthetic and hydrolyzing genes [65,66]. The truncated isoform OsbZIP58β is induced under heat stress and displays lower transactivation activity than the full-length isoform OsbZIP58α, thus fine-tuning starch accumulation and grain filling during heat stress [65].

**Figure 3:**
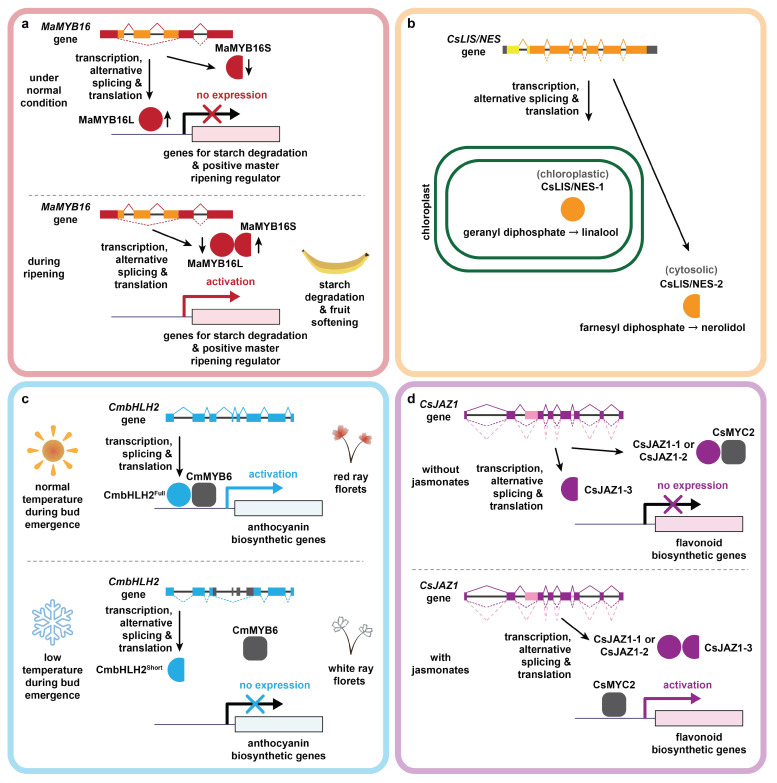
Examples of plant metabolism regulated by alternative splicing. (**a**) Regulation of transcription factor MaMYB16 in banana by alternative splicing and its roles in starch metabolism; (**b**) regulation of CsLIS/NES in tea plants by alternative splicing and its roles in linalool and nerolidol biosynthesis; (**c**) regulation of CmbHLH2 in chrysanthemums by alternative splicing and its roles in anthocyanin biosynthesis and floret coloration; (**d**) regulation of JASMONATE ZIM-domain (JAZ) repressor in tea plants by alternative splicing and its roles in jasmonate-mediated flavonoid biosynthesis.

Alternative splicing regulates fruit ripening associated with starch metabolism in banana (*Musa acuminate*) (Figure 3a) [67]. An R1-type MYB transcription factor, namely *MaMYB16*, is spliced through alternative 5′ splicing, exon skipping and alternative 3′ splicing, generating two isoforms [67]. The full-length isoform MaMYB16L binds to the promotors of genes involved in starch degradation, including isoamylases (*MaISA2*), β-amylase (*MaBAM7*) and α-amylases (*MaAMY3*), as well as positive master ripening regulator dehydration-responsive element-binding factor (*MsDREB2*), suppressing their expressions [67]. By contrast, the short isoform MaMYB16S could not bind to these promoters due to the lack of a DNA binding domain, but could bind to MaMYB16L and inhibit its DNA binding and transactivation activities [67]. During fruit ripening, *MaMYB16L* is downregulated, while *MaMYB16S* is upregulated, hence promoting starch degradation and fruit softening [67].

#### 3.2.2. Lipid Metabolism

Lipids are primary metabolites essential for plants. As a major constituent of membranes, lipids make up 5–10% dry weight of plant vegetative cells [68]. In seeds, lipid storage is an energy reserve for securing the survival of young seedlings after germination [69]. They are also consumed as food by humans and animals as well as used as biofuels in the biorefinery industry [70].

Phosphatidic acid is an intermediate for the generation of membrane lipids and storage lipids [71]. It is also involved in various cellular processes such as signal transduction in response to stimuli, secretion and membrane trafficking [71]. In plants, diacylglycerol kinase (DGK) catalyzes the conversion of diacylglycerol to phosphatidic acid [72]. Alternative splicing generates two splice isoforms of DGK in tomato (*Solanum lycopersicum*), both harboring DGK catalytic activity [72]. The full-length isoform delineated as LeCBDGK harbors a calmodulin-binding domain at the C-terminus and could bind to calmodulin [72], which is a calcium ion binding regulatory protein that could be activated by calcium ion [73]. In response to calcium ion, LeCBDGK is translocated from soluble cell fraction to membrane fraction [72]. On the other hand, the truncated isoform LeDGK1 that lacks the calmodulin-binding domain is insensitive to calcium ion [72]. Thus, alternative splicing regulates the generation of calcium-sensitive and -insensitive DGK isoforms, providing flexibility in response to calcium ion [72].

Biosynthesis of triacylglycerol, the major form of energy storage in seed oil crops, requires diacylglycerol acyltransferase (DGAT) in the Kennedy pathway [74]. In peanuts (*Arachis hypogaea*), AhDGAT1 is regulated by alternative splicing in an organ-dependent manner [75]. Except the two truncated splice isoforms AhDGAT1.2 and AhDGAT1.4, all the other five isoforms are functional and could complement the free fatty acid lethality phenotype of a triacylglycerol-deficient *Saccharomyces cerevisiae* strain [75]. However, the actual contribution and biochemical meanings of these isoforms on triacylglycerol production and storage in peanuts remain unclear.

WRINKLED1 (WRI1), which belongs to the APETALA2/ethylene-responsive element binding protein transcriptional factor family, acts as a master regulator for triacylglycerol biosynthesis in plants [76]. In castor bean (*Ricinus connunis* L.), both of the two splice isoforms, RcWRI1-A and RcWRI1-B, which differ by three amino acids in length, are functional, with RcWRI1-B appearing to be more active [77]. RcWRI1-A is expressed in all plant tissues, whereas RcWRI1-B expression is specific to seeds [77]. Thus, alternative splicing likely plays a regulatory role to improve lipid biosynthesis in castor bean seeds.

#### 3.2.3. Photorespiration

Photorespiration is a pathway to recycle the toxic product, 2-phosphoglycolate, formed when ribulose-1,5-bisphosphate carboxylase/oxygenase (RuBisCO) utilizes oxygen instead of carbon dioxide [78]. As a competitive pathway for carbon dioxide fixation that causes carbon, nitrogen and energy loss, photorespiration is a major target for bioengineering to improve plant growth and yield [78]. As the intermediates of photorespiration are also toxic, glycolate-glyoxylate metabolism exists for detoxification to maintain normal plant growth [78].

Hydroxypyruvate reductase (HPR) converts hydroxypyruvate to glycerate in glycolate-glyoxylate metabolism [79]. In pumpkin (*Cucurbita* sp.), two splice isoforms of HPR (HPR1 and HPR2) are generated by alternative 5′ splicing [79]. Compared with HPR1, HPR2 lacks a targeting sequence for peroxisome localization [79]. Accordingly, HPR2 is cytosolic, whereas HPR1 is localized in peroxisomes [79]. In darkness, HPR1 and HPR2 are both weakly expressed [79]. Under light, HPR2 but not HPR1 is strongly induced [79]. Therefore, light regulates alternative splicing of HPR with specific cellular localization. It remains unclear whether the two HPRs harbor the same catalytic ability.

#### 3.2.4. Ascorbate Metabolism

Ascorbate, a downstream metabolite of D-glucose, D-mannose and/or *myo*-inositol, is the most abundant water-soluble antioxidant in plants [80]. It scavenges and regulates the level of hydrogen peroxide, a reactive oxygen species, in plant cells by reduction in the ascorbate/glutathione cycle (or Asada–Halliwell pathway) through the action of ascorbate peroxidase (APX) [81]. Consequently, ascorbate and hydrogen peroxide are converted into monodehydroascorbate and water, respectively, and recycled in the pathway [73,74]. Due to its antioxidant activities and health-promoting effects, ascorbate is considered an important food component in human diets [82].

Early studies suggested that chloroplastic APXs are regulated by alternative splicing developmentally in some plant species [83,84]. In pumpkin, the stromal APX that lacks a putative membrane-spanning domain in the C-terminus is a splice isoform of the thylakoid-bound APX, which is formed as a consequence of alternative 3′ splicing [83]. Similarly, stromal and thylakoid-bound APXs are produced from a single gene by alternative splicing in spinach (*Spinacia oleracea*), but these isoforms are generated by intron retention and/or alternative last exon [84,85,86]. A putative splicing regulatory *cis*-element upstream of the acceptor site of intron 12 in this spinach APX was found to be crucial for the selection of splice sites, but the exact nuclear protein(s) that interact with this *cis*-element and the regulatory mechanism remain unknown [87]. Interestingly, the stromal and thylakoid-bound APXs in *Arabidopsis*, rice and tomato are encoded by separate genes [80,88,89].

Alternative splicing regulates wild emmer wheat’s (*Triticum turgidum* ssp. *Dicoccoides*) wheat kinase start1 (*WKS1*) resistance gene that confers resistance to *Puccinia striiformis* f. sp. *tritici* (*Pst*), the causal agent of stripe rust, through catalyzing phosphorylation of thylakoid-bound APX [90,91]. When inoculated with *Pst* under high temperatures, the full-length isoform WKS1 that harbors a START domain at the C-terminus is upregulated, whereas the major splice isoform WKS2 that lacks the START domain is downregulated [90,91]. WKS1 can be translocated to chloroplasts where it binds, phosphorylating the thylakoid-bound APX, reducing its activity and ability to detoxify hydrogen peroxide [90]. It was proposed that this would eventually lead to cell death after several days, considerably longer than *R*-genes-triggered hypersensitive responses which restrict pathogen growth in the host [90]. By contrast, WKS2 appears to be non-functional and is unable to bind or phosphorylate APX [90]. Hence, WKS1, but not WKS2, is a candidate isoform that confers resistance towards *Pst* and could be introduced to wheat (*Triticum aestivum*), a close relative of wild emmer wheat and a major food crop, by transgenic approaches [90].

### 3.3. Phytohormones

#### 3.3.1. Auxin Metabolism

Auxin is a major phytohormone that regulates plant growth and development by mediating cell division, elongation and differentiation [92]. Its roles in apical-basal polarity and responses to tropisms have been extensively studied [93,94]. Auxin metabolism includes biosynthesis, conjugation and degradation [92]. Our current understanding of auxin biosynthesis suggests the existence of multiple pathways in plants [95].

In the proposed auxin biosynthetic pathways, a family of YUCCA proteins, which are flavin-dependent mono-oxygenases, catalyzes the conversion of tryptamine to *N*-hydroxytryptamine in the tryptophan-dependent pathway [95,96]. As a result of alternative splicing, two isoforms of YUCCA4 are formed in *Arabidopsis* [96]. YUCCA4-1 is cytosolic and is detectable in all tissues [96]. By contrast, YUCCA4-2, which harbors a predicted C-terminal hydrophobic transmembrane domain, is inserted into the endoplasmic reticulum membrane and is specific in flowers [96]. Both isoforms harbor the expected YUCCA4 catalytic activities [96]. Taken together, alternative splicing regulates subcellular localization of YUCCA4, which may lead to compartmentation of auxin biosynthesis [96].

#### 3.3.2. Jasmonate Metabolism

Jasmonates, including jasmonic acid as well as its precursor and derivatives, are a group of phytohormones that regulate plant growth and development, especially under biotic and abiotic stresses [97]. For example, jasmonates mediate senescence and leaf abscission and inhibit seed germination [98]. Jasmonates are derived from linolenic acid and are structurally similar to eicosanoids in mammals [98].

In poplar (*Populus tomentosa*), alternative splicing fine-tunes the molecular mechanism of leaf senescence [99]. PtRD26 is a NAC transcription factor that acts as a positive regulator for leaf senescence [99]. The downstream genes that PtRD26 regulates include several senescence-associated NAC family transcription factors, proteins related to chlorophyll degradation and lysine catabolism as well as lipoxygenase 2 (LOX2) for jasmonate biosynthesis and 1-aminocyclopropane-1-carboxylic acid synthase 6 (ACS6) for ethylene biosynthesis [99]. Alternative splicing of PtRD26 by intron retention occurs during leaf senescence, generating the truncated splice isoform PtRD26^IR^ [92]. PtRD26^IR^ could interact with several senescence-associated NAC family transcription factors and repress their DNA binding affinity, resulting in the delay of age-, dark- and PtRD26-induced leaf senescence [99].

Lipoxygenase (LOX) catalyzes the oxygenation of polyunsaturated fatty acids for further jasmonate biosynthesis [100]. In tea plants, six out of eleven LOX genes are differentially regulated by alternative splicing [100]. During feeding by tea geometrids, infection by *Glomerella cingulate*, cold stress and jasmonate treatment, the corresponding CsLOX truncated isoforms are induced [100]. It was suggested that these splice isoforms might regulate LOXs by competing or compensating with the full-length isoforms [100].

### 3.4. Primary and Specialized Metabolism

#### Terpenoid Metabolism

Terpenoids, which include all compounds derived from isopentenyl diphosphate (IPP) and dimethylallyl diphosphate (DMAPP), are a large family of primary and specialized metabolites found in all living organisms [101,102]. They are structurally diverse compounds commonly categorized as hemiterpene (C_5_), monoterpenes (C_10_), sesquiterpenes (C_15_), diterpenes (C_20_), sesterterpenes (C_25_), triterpenes (C_30_) and tetraterpenes (C_40_) [103,104]. Terpenoids have diverse functions in plants. For example, they act as phytohormones [105] and also serve as signals for attracting pollinators [106], avoiding herbivores [107] and mediating interactions among plants [108]. Terpenoids have a wide range of applications in human diets and the food industry. Volatile terpenoids such as linalool and nerolidol contribute to the odor of teas [109,110]. Terpenoids including bixin, lycopene and astaxanthin are widely used as colorants in the food industry [111]. Terpenoids in herbs and spices help in preserving food due to their microbicidal and insecticidal activities [112]. Some terpenoids also harbor pharmaceutical and health-promoting effects against cancer, inflammation and infectious diseases [103,104].

Various transcriptomic studies have revealed that terpenoid biosynthetic genes are regulated by alternative splicing in different tissues and stress conditions. For instance, terpenoid biosynthetic genes are regulated by alternative splicing in different tissues of *Ginkgo biloba* [113], Sichuan pepper (*Zanthoxylum armatum*) [114], *Artemisia argyi* [115] and *Lindera glauca* [116]. They are also alternatively spliced under drought and heat stress in tea plants [117].

Terpene synthases are a family of enzymes catalyzing the committed steps for generating isoprene, monoterpenes, sesquiterpenes, diterpenes and triterpenes [102,118]. In Dong Ling Cao (*Isodon rubescens*), alternative 3′ splicing of a type I terpene synthase *IrKSL3* generates two splice isoforms with different biochemical activities [119]. Using copalyl diphosphate as a substrate, the full-length IrKSL3 produces miltiradiene as the sole product, whereas the splice isoform IrKSL3a that harbors a deletion of six amino acids could simultaneously generate isopimaradiene and miltiradiene [119]. These results illustrate that alternative splicing could influence product outcomes in enzyme-catalyzed reactions [119].

Biosynthesis of linalool and nerolidol also requires terpene synthase [120]. Two splice isoforms of terpene synthase could be detected in tea plants (Figure 3b) [120]. The full-length isoform CsLIS/NES-1 is localized in chloroplasts, functioning as a linalool synthase [120]. The splice isoform CsLIS/NES-2 with an N-terminal truncation is cytosolic, acting as a nerolidol synthase [120]. As both isoforms are bifunctional in catalyzing the in vitro generation of linalool and nerolidol from geranyl diphosphate and farnesyl diphosphate, respectively, the difference in their subcellular localizations likely contributes to the discrepancy of their *in planta* biochemical functions [120]. In addition, expression of *CsLIS/NES-1* and *CsLIS/NES-2* was differentially regulated [120]. *CsLIS/NES-1* is induced by jasmonates, whereas *CsLIS/NES-2* expression level is higher in flowers than in leaves [120].

Phylloquinone, which is also known as vitamin K_1_, is a prenylated naphthoquinone [121]. In most photosynthetic plants, phylloquinone serves as an electron acceptor in photosystem I [122] and an electron carrier for disulfide bond formation in proteins essential for photosystem II assembly [123]. Dietary consumption of phylloquinone is beneficial for human health due to its roles in maintenance of bones [124], blood coagulation [125] and prevention of cardiovascular diseases [126]. Isochorismate synthase (ICS) converts chorismate from the shikimate pathway to isochorismate, a key intermediate for the biosynthesis of phylloquinone [127]. Poplar *(Populus trichocarpa) ICS* undergoes extensive alternative splicing, producing at least 37 splice isoforms that represent approximately 50% of total *ICS* transcripts [128]. Most splice isoforms are formed from intron retention and/or alternative 3′ splicing and harbor premature stop codons [128]. This is in contrast to *Arabidopsis AtICS1* that predominantly generates a full-length transcript [128]. Accordingly, it was proposed that alternative splicing of *ICS* was recruited independently during evolution [128]. *Populus* ICS mainly functions in phylloquinone biosynthesis, which can be maintained at a low functional transcript level [128], whereas *Arabidopsis* AtICS1 is predominantly involved in the biosynthesis of stress-induced salicylic acid, which could also be synthesized from isochorismate [129].

### 3.5. Specialized Metabolism

#### 3.5.1. Alkaloid Metabolism

Alkaloids are specialized metabolites that harbor at least one nitrogen atom in their heterocyclic rings [130,131]. They could be further classified into different groups according to their backbone structures [131]. Most of them are generated from amino acids: phenylalanine, tyrosine, tryptophan and ornithine [132]. In plants, some alkaloids are toxins involved in defense against pathogens [133], insects [134] and herbivores [135]. Owing to their excellent toxicity and pharmaceutical activities, alkaloids have been extensively exploited for poisoning and medical uses [136,137]. Well-known examples of alkaloids include caffeine, morphine, strychnine and nicotine.

Monoterpene indole alkaloids, a group of alkaloids generated from the combination of tryptophan and terpenoid precursors, are mainly distributed in Apocynaceae, Loganiaceae and Rubiaceae [138]. To generate the active substrate for biosynthesis of monoterpene indole alkaloids, strictosidine β-D-glucosidase (SGD) catalyzes deglycosylation of strictosidine to release the highly reactive aglycone [139]. In Madagascar periwinkle (*Catharanthus roseus*), alternative last exon splicing generates a full-length isoform SGD with glucosidase activities and another isoform shSGD harboring a truncated C-terminus and lacking glucosidase activities [139]. shSGD interacts with SGD and disrupts multimerization of SGDs, which, in turn, inhibits the SGD deglycosylation activities [139]. Thus, generation of a pseudo-enzyme by alternative splicing of SGD serves as a regulatory mechanism to fine-tune monoterpene indole alkaloid biosynthesis [139].

Allantoin is a nitrogen-rich ureide compound generated from degradation of purines [140]. In plants, its biosynthesis requires transthyretin-like (TTL) protein [140]. The two isoforms of TTL in *Arabidopsis*, which are generated by alternative 3′ splicing, harbor similar in vitro catalytic activities with different subcellular localizations [140]. An internal peroxisomal targeting signal present in the long isoform TTL^1−^ is missing from the short isoform TTL^2−^ [140]. Exploration of TTLs in other plant species suggests that alternative splicing of internal peroxisomal targeting signal appears to be a conserved regulatory mechanism in angiosperm [140].

#### 3.5.2. Phenylpropanoid Metabolism

Phenylpropanoids and their downstream metabolites such as flavonoids, monolignols/lignin, stilbenoids, lignans and suberin are phenolic compounds derived from the amino acids phenylalanine and/or tyrosine [141,142]. Their biosynthesis and regulation have been extensively studied due to their functions in cell wall structure [143,144], anti-oxidation [145,146], UV protection [147,148], determination of fruit and flower colors [149,150], defense against pathogens [151,152] and herbivores [153,154] and fertility [155,156], as well as their contribution to human activities such as biomass utilization [157], nutrition [158] and breeding [159]. Depending on the types of phenylpropanoids and plant species, some of the phenylpropanoids are constitutively accumulated, while some of them are induced by stresses [151]. Their accumulation is often correlated to stress tolerances [151].

Biosynthetic genes in phenylpropanoid and its downstream pathways are regulated by alternative splicing during plant development. For example, in kiwifruit (*Actinidia chinensis*), several structural genes for anthocyanin biosynthesis, including chalcone synthase (*CHS*), flavanone 3-hydroxylase (*F3H*), dihydroflavonol-4-reductase (*DFR*), anthocyanidin synthase (*ANS*) and uridine diphosphate (UDP)-glucosyltransferase (*UGT*) are regulated by alternative splicing during fruit development and ripening [160]. In tea plants, a series of genes involved in phenylpropanoid, anthocyanin and monolignol biosynthesis is also alternatively spliced in cultivars that harbor purple or green tender shoots [161], throughout leaf development [162] and in different tissues [81]. Similar observations were found in *G. biloba*, in which alternative splicing regulates flavonoid biosynthetic genes and their transcription regulators in different tissues [163].

Alternative splicing also regulates phenylpropanoid and its downstream pathway genes during stress conditions. In sorghum (*Sorghum bicolor*), upregulation and alternative splicing of flavonoid and phenylpropanoid biosynthetic genes occur upon infection of *Colletotrichum sublineola*, the causal agent of anthracnose [65]. In *Arabidopsis*, phenylpropanoid biosynthetic genes such as phenylalanine ammonia lyases (*PAL*) and 4-coumarate CoA ligases (*4CL*) as well as the downstream monolignol biosynthetic genes such as hydroxycinnamoyl-CoA shikimate/quinate hydroxycinnamoyltransferase (*HCT*) and coniferaldehyde 5-hydroxylase (*CAld5H*) are alternatively spliced during iron deficiency [51]. These are expected to be related to the excretion of phenylpropanoids, which could chelate iron, to the rhizosphere [51]. Collectively, these works suggest that alternative splicing potentially plays crucial roles in mediating phenylpropanoid biosynthesis in plants under both biotic and abiotic stresses.

Alternative splicing is a regulatory mechanism that governs anthocyanin production and floret colors in some plant species under different conditions. For instance, temperatures during flower bud emergence affect anthocyanin biosynthesis and floret coloration in chrysanthemums (*Chrysanthemum morifolium*) [164]. The temperature-dependent coloration was found to be attributed by alternative splicing of a basic helix–hoop–helix transcription factor gene *CmbHLH2* (Figure 3c) [164]. The red ray florets generate a full-length functional CmbHLH2^Full^, whereas the white ray florets produce a truncated CmbHLH2^Short^ due to alternative position and exon skipping [164]. Unlike CmbHLH2^Full^, CmbHLH2^Short^ fails to interact with the MYB transcription factor CmMYB6 or activate anthocyanin biosynthetic genes [164]. A similar example was reported in peach (*Prunus persica*), which produces flowers of different colors on the same tree [165]. It was found that white flowers generate a truncated non-functional ANS by alternative splicing [165]. Hence, different cultivation conditions induce alternative splicing of the key genes for anthocyanin biosynthesis, thereby impacting floret colors.

Anthocyanin biosynthesis in higher plants is typically activated by the MBW (MYB–basic helix-loop-helix protein–WD40 repeat; MYB–bHLH–WDR) protein complexes [166]. In rapeseed (*Brassica napus* L.), BnaPAP2 is an MYB transcription factor required for regulating anthocyanin biosynthesis [167]. Alternative splicing generates different BnaPAP2.A7 isoforms with opposite functions [167]. The full-length BnaPAP2.A7-744 harbors all the essential domains of MYB and could interact with a bHLH protein in vitro [167]. The splice isoforms BnaPAP2.A7-910 and BnaPAP2.A7-395 are truncated and cannot interact with bHLH proteins [167]. Although the exact molecular mechanism is still unknown, BnaPAP2.A7-910 and BnaPAP2.A7-395 downregulate flavonoid biosynthetic genes when overexpressed in *Arabidopsis*, suggesting that their roles as suppressors are in opposition to the function of the full-length BnaPAP2.A7-744 as an activator [167]. Thus, alternative splicing provides a mechanism to balance the positive and negative regulations of anthocyanin biosynthesis in rapeseed.

JASMONATE ZIM-domain (*JAZ*) repressor, a negative regulator of diverse jasmonate responses including flavonoid biosynthesis [168], is regulated by alternative splicing in tea plants which produces three JAZ splice isoforms (Figure 3d) [169]. The full-length isoform CsJAZ1-1 is localized in nucleus, whereas the truncated isoforms CsJAZ1-2 and CsJAZ1-3, which lack the 3′ coding sequences, are localized in both nucleus and cytoplasm [169]. In the absence of jasmonates, CsJAZ1-1 and CsJAZ1-2, but not CsJAZ1-3, competitively bind to CsMYC2, a positive regulator of flavonoid biosynthesis [170], thereby inactivating CsMYC2 and repressing flavonoid biosynthetic genes such as dihydroflavonol reductase (*DFR*) and leucoanthocyanidin dioxygenase (*LDOX*) [169]. In the presence of jasmonates, CsJAZ1-3 interacts with CsJAZ1-1 and CsJAZ1-2, preventing their binding to CsMYC2 and eventually leading to their degradation. Consequently, repression of CsMYC2 and flavonoid biosynthesis was released [169]. Collectively, alternative splicing coordinately regulates jasmonate-mediated flavonoid biosynthesis in tea plants [169].

**Table 1:** Genes related to plant metabolism that are regulated by alternative splicing.

Type of Metabolism	Metabolic Pathways	Species	Gene Alternatively Spliced	Spliced Isoforms and Their Functions	References
Primary metabolism	Starch metabolism	*Phaseolus vulgaris* L.	Starch-branching enzyme (SBE)	LF-PvSBE2: long form, targeted to starch granule and cytosol	[37]
				PvSBE: truncated, targeted to cytosol	
		Arabidopsis (*Arabidopsis thaliana*)	Indeterminate domain 14 (IDD14)	IDD14α: full-length, activates *Qua-Quine Starch* (*QQS*)	[63]
				IDD14β: truncated, lacks DNA binding domains, inhibits DNA binding ability of IDD14α	
		Rice (*Oryza sativa*)	OsbZIP58	OsbZIP58: full-length, mediates grain filling by regulating the expression of starch biosynthetic and hydrolyzing genes	[65]
				OsbZIP58β: induced under heat stress, displayed a lower transactivation activity than the full-length isoform OsbZIP58α	
		Banana (*Musa acuminate*)	MaMYB16L	MaMYB16L: full-length, binds to the promotors and activates genes involved in starch degradation	[67]
				MaMYB16S: truncated, binds to MaMYB16L, inhibits its DNA binding and transactivation activities	
	Lipid metabolism	Tomato (*Solanum lycopersicum*)	Diacylglycerol kinase (DGK)	LeCBDGK: full-length, harbors DGK catalytic activity, harbors a calmodulin-binding domain, could bind to calmodulin	[72]
				LeDGK1: truncated, harbors DGK catalytic activity, lacks a calmodulin-binding domain, could not bind to calmodulin	
		Peanuts (*Arachis hypogaea*)	Diacylglycerol acyltransferase (DGAT)	AhDGAT1.1, AhDGAT1.3, AhDGAT1.5, AhDGAT1.6 and AhDGAT1.7: harbor DGAT activities	[75]
				AhDGAT1.2 and AhDGAT1.4: truncated, lack DGAT activities	
		Castor bean (*Ricinus connunis* L.)	WRINKLED1 (WRI1)	RcWRI1-A: functional, less active, is expressed in all tissues	[77]
				RcWRI1-B: functional, more active, expression specific to seeds	
	Photorespiration	Pumpkin (*Cucurbita* sp.)	Hydroxypyruvate reductase (HPR)	HPR1: full-length, harbors a targeting sequence for peroxisome localization, localized in peroxisomes, induced under light	[79]
				HPR1: truncated, lacks a targeting sequence for peroxisome localization, localized in cytosol, weakly expressed in dark and under light	
	Ascorbate metabolism	Pumpkin (*Cucurbita* sp.)	Ascorbate peroxidase (APX)	Thylakoid-bound APX: harbors a putative membrane- spanning domain in the C-terminus, localized in thylakoid	[83]
				Stromal APX: lacks a putative membrane-spanning domain in the C-terminus, localized in stroma	
		Spinach (*Spinacia oleracea*)	Ascorbate peroxidase (APX)	Thylakoid-bound APX: harbors a putative membrane- spanning domain in the C-terminus, localized in thylakoid	[84,85,86]
				Stromal APX: lacks a putative membrane-spanning domain in the C-terminus, localized in stroma	
		Wheat (*Triticum turgidum* ssp. *Dicoccoides*)	Wheat kinase start1 (WKS1) resistance gene	WKS1: full-length, harbors a START domain at the C-terminus, upregulated under high temperature and when inoculated with *Pst*, translocated to chloroplast, binds, phosphorylates and reduces the activity of thylakoid-bound APX	[90]
				WKS2: lacks the START domain, downregulated under high temperature and when inoculated with *Pst*, non-functional, unable to bind or phosphorylate APX	
Phytohormone	Auxin metabolism	*Arabidopsis* (*Arabidopsis thaliana*)	Flavin-dependent mono-oxygenase (YUCCA4)	YUCCA4-1: lacks a predicted C-terminus hydrophobic transmembrane domain cytosolic, expressed in all tissues	[96]
				YUCCA4-2: harbors a predicted C-terminus hydrophobic transmembrane domain, inserted into endoplasmic reticulum membrane; expressed in flowers	
	Jasmonate metabolism	Poplar (*Populus tomentosa*)	NAC transcription factor (PtRD26)	PtRD26: full-length, activates several senescence-associated NAC family transcription factors, proteins related to chlorophyll degradation, lysine catabolism, lipoxygenase 2 (LOX2) for jasmonate biosynthesis and 1-aminocyclopropane-1-carboxylic acid synthase 6 (ACS6) for ethylene biosynthesis	[99]
				PtRD26: truncated, interacts with several senescence-associated NAC family transcription factors and represses their DNA binding affinity	
		Tea plants (*Camellia sinensis*)	Lipoxygenase (LOX)	Full-length isoform: predominant during normal conditions	[100]
				Truncated splice isoforms: induced during feeding by tea geometrids, infection by *Glomerella cingulate*, cold stress and jasmonate treatment	
Primary and Specialized metabolism	Terpenoid metabolism	Dong Ling Cao (*Isodon rubescens*)	Terpene synthase (IrKSL3)	IrKSL3: full-length, produces miltiradiene as the sole product from copalyl diphosphate	[119]
				IrKSL3a: shorter, simultaneously generates isopimaradiene and miltiradiene from copalyl diphosphate	
		Tea plants (*Camellia sinensis*)	Terpene synthase (LIS/NES)	CsLIS/NES-1: full-length, localized in chloroplast, functions as a linalool synthase, induced by jasmonates	[120]
				CsLIS/NES-2: harbors a truncated N-terminus, localized in cytosol, functions as a nerolidol synthase, expression is higher in flowers than in leaves	
		Poplar (*Populus trichocarpa*)	Isochorismate synthase (ICS)	*Populus ICS* undergoes extensive alternative splicing, produces at least 37 splice isoforms that represent approximately 50% of total *ICS* transcripts	[128]
Specialized metabolism	Alkaloid metabolism	Madagascar periwinkle (*Catharanthus roseus*)	Stictosidine β-D-glucosidase (SGD)	SGD: full-length, harbors glucosidase activities	[139]
				shSGD: harbors a truncated C-terminus, lacks glucosidase activities, interacts with SGD, disrupts multimerization of SGD, inhibits deglycosylation activities of SGD	
		*Arabidopsis* (*Arabidopsis thaliana*)	Transthyretin-like (TTL) protein	TTL^1−^: long isoform, harbors an internal peroxisomal targeting signal	[140]
				TTL^2−^: short isoform, lacks an internal peroxisomal targeting signal	
	Phenylpropanoid metabolism	Chrysanthemum (*Chrysanthemum morifolium*)	Basic helix–hoop–helix transcription factor (CmbHLH2)	CmbHLH2^Full^: full-length, expressed in red ray florets, interacts with CmMYB6 and activates anthocyanin biosynthetic genes	[164]
				CmbHLH2^Short^: truncated, expressed in white ray florets, cannot interact with CmMYB6 or activate anthocyanin biosynthetic genes	
		Peach (*Prunus persica*)	Anthocyanidin synthase (ANS)	Full-length ANS: functional, generates red flowers	[165]
				Truncated ANS: non-functional, generates white flowers	
		Rapeseed (*Brassica napus* L.)	MYB transcription factor (BnaPAP2)	BnaPAP2.A7-744: full-length, harbors all the essential domains of MYB, could interact with bHLH protein, activates flavonoid biosynthetic genes	[167]
				BnaPAP2.A7-910 and BnaPAP2.A7-395: truncated, cannot interact with bHLH protein, downregulates flavonoid biosynthetic genes	
		Tea plants (*Camellia sinensis*)	JASMONATE ZIM-domain (JAZ) repressor	CsJAZ1-1 and CsJAZ1-2: full-length (CsJAZ1-1) and truncated (CsJAZ1-2), bind to CsMYB2, resulting in inactivation of flavonoid biosynthetic genes	[169]
				CsJAZ1-3: truncated, binds to CsJAZ1-1 and CsJAZ1-2 in the presence of jasmonates and prevents their binding to CsMYB2, resulting in activation of flavonoid biosynthetic genes	

## 4. Conclusions and Future Directions

Owing to the advancement of high-throughput third-generation RNA sequencing technique, omics analyses and related technology in the past decades, full-length transcripts can be efficiently obtained, providing valuable information regarding regulation of genes by alternative splicing [171,172]. Thus far, a diverse array of genes in plant metabolism that are regulated by alternative splicing has been determined in different developmental stages and/or stress conditions. Some specific examples of how alternative splicing fine-tunes various metabolic pathways in plants by altering biochemical activities, interaction and/or subcellular locations of proteins encoded by different splice isoforms of genes have also been provided. Altogether, these findings could advance our understanding of post-transcriptional regulation of plant metabolism for coping with stresses and modulating growth and development.

Remaining tasks ahead include functional characterization of all alternatively spliced isoforms for each gene and determination of their involvement in regulating different metabolic processes. Meanwhile, conservation of alternative splicing of a particular gene in metabolic processes in the plant kingdom, if any, and to what extent, may indicate evolutionary significance of metabolic pathways and reveal mechanisms underlying alternative splicing. Furthermore, it is known that epigenetic modifications, such as DNA methylation, regulate alternative splicing in animals [173]. It has also been shown recently that mRNA methylation, such as methylation of adenosine at the N6 position (m^6^A), alters the occurrence of alternative splicing events and expression of splice isoforms in *Arabidopsis* [174]. Currently, it remains to be examined whether epigenetic modification serves as another level of regulation for plant metabolism through mediating alternative splicing.

Studies of the roles of alternative splicing in regulating plant metabolism will provide essential foundational information that could open up new avenues for bioengineering. Current approaches of overexpression, downregulation and knockout mutation usually only consider the predominant splice isoforms of target genes available in public databases. With the knowledge of different splice isoforms, including their differences in biological and biochemical functions and/or subcellular localization, the potential of bioengineering could be largely extended through overexpression, downregulation or knockout via CRISPR/cas9-mediated mutagenesis of specific isoforms of a gene of interest, thus fine-tuning the desired metabolic processes in plants. In fact, specific suppression of targeted splice isoforms of SGD and shSGD was successfully achieved in *C. roseus* by virus-induced gene silencing previously [139]. Overall, these will provide new insights into improvement of plant performance, yield and utility through bioengineering.

## Data Availability

Not applicable.
